# 

**Published:** 1891-10-03

**Authors:** 


### The Hospital

A Weekly Institutional Journal of

### Science, Medicine, Nursing, and Philanthropy.

EDITED BY

HENRY C. BURDETT,

AUTHOR OF "HOSPITALS AND ASYLUMS OF THE WORLD: THEIR ORIGIN, HISTORY, CONSTRUCTION, ADMINISTRATION, MANAGEMENT, AND LEGISLATION"; "HOSPITALS AND THE STATE"; "PAY HOSPITALS OF THE WORLD"; "COTTAGE HOSPITALS, GENERAL, FEVER, AND CONVALESCENT, WITH FIFTY BEDS AND UNDER": "BURDETT'S HOSPITAL ANNUAL AND YEARBOOK OF PHILANTHROPY"; AND "HELPS TO HEALTH."

14154

ACTING EDITOR

GEORGE W. POTTER, M.D.,

AUTHOR OF "MINISTERING WOMEN"; AND NUMEROUS PAPERS ON SCIENTIFIC AND MEDICAL SUBJECTS.

INDEX TO VOL. XI.

(3RD OCTOBER, 1891, TO 26TH MARCH, 1892.)

FOR 1891—92.

LONDON:

PUBLISHED BY THE SCIENTIFIC PRESS (LIMITED), 140, STRAND, W.C.

W1
H069X

### INDEX TO VOL. XI. 1891-92.

Abdominal Tuberculosis, 282
Aberdeen City Hospital, 218
—— Cottage Hospital, 6
—— Infirmary, 314
Accounts, A Uniform System of, 223, 224
Acrobats, Child, 256
Addenbrooke's Hospital, 42
Adulteration of Drugs, 15
African Philanthropy, 8
Agricultural Depression and Hospitals, 262
Albert and Victoria Society, The, 122
Alcock, Sir Rutherford, 244
Already! 309
Ambulance Service, London Street, 278
America and Her Hospital Workers, 85
American Hospitals, 125, 181, 193, 205, 217, 228, 248, 253, 276
—— Sensation, An, 285
Anæmia, 185
Antimony, 12, 306
Antiseptic Treatment of Scarlet Fever, 16
—— and Aseptic Surgery, 130
Antivivisection Memorial, The, 73
Appeals, 226
Arcana Fairfaxiana, 95
Army Medical Department, The, 206
Arteries, Capillaries, and Veins, Diseases of the, 185, 197
Australia, Cottage Hospitals in, 6

Baby Carrier, The, 47
Bacteriology, 20, 292
Benevolence, Cheap, 38
Biniodide of Mercury, 5
Birmingham Hospital Saturday Fund, 18, 166
—— New General Hospital, 302
—— Water Supply, 8
Blackmail on Hospital Secretaries, 126
Blepharospasm, 209
Boscombe Hospital, 194
Bowlan, Dr., Presentation to, 102
British Medical Association, The, 11, 132
"British Medical Journal," The, and the Hospitals, 194
Breakers Ahead! 238
Bristol Eye Hospital, 254
Burial, The Ordeal of, 202

Canadian Hospitals, 76
Cancer Cure, A, 208
Cardinals, Two English, 119
Carpenters' Company, The, and the Sanitary Institute, 128
Caterers, An Exhibition of, 71
Cats, 213
Cerebral Hæmorrhage in the Aged, 117
Charing Cross Hospital, 114, 266
Charity—Limited, 274
—— Organization Society, The, 182
Chemists, Congress of Analytical, 30
Chemist's Profession, The, 80
Cheshire County Asylum, 152
Chest, Royal Hospital for Diseases of the, 114
Chester County Asylum, 212
Children, Feeble-minded, 292
Chloroform and Ether, 293
Christianity, Editorship and Nurses, 263
Christmas at the Hospitals, 141
Church Congress, Science at the, 27
—— Death in the, 237
Cigarette Smoking, 182
Cirrhosis of the Liver, 318
City of London Lying-in Hospital, 154
—— Truss Society, 230
Clapham Maternity Hospital, 66
Clarence, Death of the Duke of, 194, 201
Cleghorn, Deputy-Surgeon-General, 6
Coldstream Cottage Hospital, 218
Coleraine Cottage Hospital, 6
Collie, Dr., 5, 42
Confusion worse Confounded, 13
Conjunctival Sac, Foreign Bodies in the, 283
Consumption, Hospitals, 218
—— Inherited, 210
Cornea, Phlyctenular Ulceration of the, 222
Criminal Anthropology, 280
Cross, Mr. G. A., on the Exemption of Charities from Rates, 154
Crusaders, A Call for, 184
Cumberland Asylum, 212

Death in the Church, 237
Dementia, 252
Denver Hospital, 205
Derby County Asylum, 236
—— Infirmary, 166, 254
Detectives, Social, 179
Devonshire Hospital and Buxton Bath Charity, 254
Diabetes, 61
Digitalis in Pneumonia, 35
Diphtheria, 5, 25, 35, 129, 294, 317
Dispensing, Practical, 171
Doctors and Lawyers, 219
Doctor's Armchair, The :—Ouida against the Physiologists, 3; Scientific Scoundrelism, 15; Science at the Church Congress, 27; Theosophy and Mrs. Besant, 39; The Cambridge Executioner, 63; Old Doctors or New, 75; The Sick in Uncivilised Lands, 87; The Woman or the Man? 99; Social Detectives, 179; Writers' Brains, 191; Are all Writers Men of Genius? 203; No Faith in Physic, 215; The Farming of Patients, 240; Ungrateful Patients, 251; Christianity, Editorship and Nurses, 263; Payn on Physic, 275; A Persecuted Hospital, 287; Hypnotism in America, 299; Recuperation, 311
Doctor's Christmas Box, The, 177
Doctors' Fees, 80
Donegal Industrial Fund, 267
Dorset County Asylum, 96
Drink Bill, The National, 268
Dublin Hospitals' Annual Inspection, 66
—— Hospital Sunday Fund, 102
Dunster and Minehead Village Hospital, 254
Dyspepsia, 93

Eastern Fever Hospital, The, 5, 18, 42
Eczema in Paddington Infirmary, 121
Edinburgh Royal Infirmary, 206, 230, 278
Electricity, Execution by, 286
—— Up to Date, 200
Enfield Isolation Hospital, 90
Epidemic, The, Preventive Measures, 213
Epidemics, 219
Epilepsy, 227
Ether and Chloroform, 293
Eucalyptus, 156
Exeunt, 286
Exostoses, Multiple Cancellous, 170
Eye Complications in Influenza, 234
—— The Effects of Anæmia on the, 185

Factory Inspectors, Female, 24
Feeble-Minded Children, The Care of, 292
Feelings and Beliefs, 109
Ferns, 4
Festival Season, The, 298
Feverfew, 288
Fires in Hospitals, 230
Flushing, 70
Fogs, 250, 256
Folklore in Congress, 20
Food and Cookery Exhibition, The, 114
—— Talks About, 17, 36, 296, 308, 320.
Fortune, A Pillar of, 44
Funds and Fairplay for the Voluntary Hospitals, 173
Funeral Reform, 126
Funerals, The Risks of, 219

Garfield Memorial Hospital, Washington, 253
Gastric Dyspepsia, 233
—— Hyperæsthesia and Acidity, 257
General Medical Council, The, 116
Genius, 203, 219
—— and Insanity, 92
Genu Valgum, 10
Glasgow Royal Infirmary, 42
Gold Against Alcohol, 316
Gout, 105
Governesses' Convalescent Home, 42
Great Northern Central Hospital, The, 80, 278
Greek, The Study of, 63
Guy's Hospital, 262

Hampstead Home Hospital, 154
Hanbury General Hospital, 106
Harveian Oration, The, 44
Harvest of the Sick Poor, The, 190
Hastings and East Sussex Hospital, The, 182, 206
Health and Holiness, 156
—— Schools, 211
Heart Disease, Cactus in, 157
Herbal Simples, The History and Capabilities of: XXXVII., Ferns, 4
XXXVIII., Feverfew, 288
Hereford County Asylum, 212
Hip-joint, Excision of the, 198
Hips, Congenital Displacement of the, 245
Home Hospitals for Jewish Incurables, The, 194
Hospital Accounts, Uniform, 223
"Hospital Annual," The, 48
Hospital Reform, 160
—— Religion, 20
—— Saturday Fund, The, 18, 160, 263, 272
—— Sunday Fund, The, 102, 155, 224, 235
—— Work Abroad, 313
Hospitals Association, The, 242
—— London Voluntary, and their Critics, 161
—— of the North-West, 76, 89
—— Situation of, 104
—— The Chronic Indigence of, 242
Hull Hospital Sunday Fund, 254
Hydrophobia, 32, 35, 66
Hypnotism, 156

Inebriety and the Legislature, 247
Influenza, 168, 189, 196, 208, 213, 225, 244, 302
—— in Australasia, 35
Inoculation, 104
Insane, Story of the, from Year to Year, 96, 152, 212, 236
Insanity, Some Scientific Aspects of, 40, 88, 101, 112, 123, 151, 180, 192, 204, 216, 227, 239, 252, 264, 284, 288, 301, 312
"Inside and Out," 110
Intubation of the Larynx, 23, 246
Invalid Children's Association, 30
Irish Dispensary Doctors, 230
—— Hospitals, 6
Iritis, 131
"Is it English?" 149
Isolation Hospitals, 172

Japan, Letters from, 123, 153, 229, 264, 300
Johns Hopkins Hospital, Baltimore, 100, 111
Jokes of Serious People, The, 162
Journalism, Popular, 150

Kettering Hospital, 254
Koch's Cure, Dr., 217

Lady Smokers, 196, 232
Larders, Dirty, 316
Lawyers and Doctors, 219
Lay Managers of Hospitals, 1
Leech's Life, 116
Leeds. Lead Poisoning in, 66
Lepers, 6
Lifeboats, 98
Liverpool Royal Infirmary, 16, 266
London, Report of the Medical Officer of Health for the Port of, 290
——— Homœopathic Hospital, The, 194
——— Hospital, The, 114, 154, 279, 286, 287
——— Hospitals and their Critics, 161
——— Unpractical, 68
——— Without Hospitals, 133
Lords' Committee, The, 78
Lymphangitis, 71

Mackenzie Sir Morell, Memorial to, 314
Manchester Royal Infirmary, The, 42
Mania, 192
Manning, Cardinal, 194, 208
Mather, Mr., M.P., and Hospitals, 310, 314
Massachusetts General Hospital, Boston, 277
Mastitis, Acute, 186
Maupassant, M. de, 191
Medical Charities, 316
——— Digest, The, 83
——— History, 64
——— Politics, 37
——— Profession, The, 30
——— Progress, 297
Medicine Hat, 77
Melancholia, 180
Metropolitan Asylums Board and the Public Health Act, 314
——— Hospital, The, 266
Middlesex Hospital, 142
Midwifery, 199
Military Hospitals, American, 217
Miners' Holiday, The, 304
Model Hospital, A, 106, 119
Monomania, 216
Morgan Commemoration Fund, 255
Morley Convalescent Home, 66
Murderer, A Boy, 292
Music, The Therapeutics of, 26
Mydriatics, 81
Myxœdema, Hybrid Grafting in, 118

National Dental Hospital, The, 314
——— Health Society, The, 30
Neurotic Novelists, 304
New and the Old, The, 309
Newcastle, Hospital Sunday at
Newcastle-on-Tyne Home for Incurables, 126
New Hospital for Women, The, 314
Norfolk County Asylum, 236
North-West London Hospital, The, 206
Northampton County Asylum, 96
Northumberland County Asylum, 212

Nottingham County Asylum, 152
Norwood Cottage Hospital, 218
Nurses: Shall they Cease to be Laywomen? 2, 14

Obesity, 46
Obscurantism, 178, 188
Obstetrics and Gynæcology, International Congress of, 302
Œdema, 281
Old Doctors or New? 103
Opacities of the Cornea, 158
Ophthalmia Neonatorum, 47
Opium, 278, 280
Ouida against the Physiologists, 3
"Our Unseen Foes," 20
Out-Patients, 266
Ovaries, Removal of the, 131
Overlapping, 273
Oxford Radcliffe Infirmary, 196, 218, 230
Oxygen, 304

Paddington Green Children's Hospital, 166
"Pall Mall Gazette," The, and the Hospitals, 149, 150, 161, 176
Papillomatous Ovarian Growths, 157
Parke, Surgeon, 68
Paraffin Lamps, 302
Paralysis, 239
Pasteur, 35
Peace or War? 1
Peerless Peer, A, 184
"Pegging Away," 226
Pennsylvania Hospital, 248
Phthisis, 9, 21, 33, 45, 69, 81
Physical Education, 234
Physiologists and Small Holdings, 268
Pneumonia, 35
Poisons, The Scheduling of, 104
——— and Poisoners, III.—Antimony—Dr. Pritchard, 12
Poplar Hospital for Accidents, 126
Politics, Medical, 37
Practical Man, The, 44
Prescriptions, 30
Press, The, and the Hospitals, 173
Prestwich Asylum, 236
Princess Alice Memorial Hospital, 254
——— of Wales, The, and her Son, 140
Proportionate Giving, 142
Professional Witnesses, 268
Public Health Act, The, 314
Puzzle, A, 65

Radcliffe Infirmary, Oxford, 196, 218, 230
Rate Supported Hospitals, 310, 314
Rates, Exemption of Charities from, 154
Rawlings, Mr. B. Burford, on Hospital Indigence, 218, 242
Reading for Children, 218
Reflection, A, 213
Registration of Nurses, The, 244
Religion, Popular, A Weak Spot in, 97
Religious Foolishness, 232
Rhus Poisoning, 269
Royal Free Hospital, 278
——— Hospital for Women and Children, The, 302
——— London Ophthalmic Hospital, The, 314
Russia, A Peep At, 28, 83
Russian Famine Fund, The, 74

St. Bartholomew's Hospital, 78
— Helen's Cottage Hospital, 6
— John (Free) Convalescent Home, 206
Salford Royal Hospital, 66
Salol in Typhoid Fever, 11
Salophene, 306
Salt Lake City Hospitals, 193
Salvationism and the Cure of the Sick, 249
"Save Us from Our Friends," 98
Sarcoma of Bone, 319
Scarlet Fever, 5
School Girls, The Bloodlessness of, 304
Scientific Scoundrelism, 15
Science, the Advance of, 309
Seamen's Hospital Society, The, 30, 166, 194, 290
Septic Thrombosis, 187
Sewage, The Disposal of, 68
Shall Nurses Cease to be Laywomen? 2, 14
Shop Assistants, 280, 290
Slave Trade, The, 184
Small-pox, 290
Smith, The Late Mr. W. H., 32
Smoke Nuisance, The, 290
Smokers, Lady, 196
Smoking, 259
Spurgeon, Mr., The late, 232
"Stable Door and the Stolen Steed, The," 225
Stertor Apoplexy and the Management of the Apoplectic State, 23
Stirling District Asylum, 42
Styptic, On a new, 221
Suppurative Keratitis, 270
Surgery, Golden Rules in, 32
Surgical Aid Society, The, 154
Survivors, 86
Syphilis, 72

Tetanus, 34
"That Poor Worm the Doctor," 80
Thomson, Sir William, 184
Theosophy, 39, 65
Thrombosis, Septic, 169
Three Weeks in a Hospital, 200
Thyroid, On Some Affections of the, 22
Ticket System, The, 259
Tipping the Porter, 62
Toronto, Medicine and Surgery in, 128
Tubercular Hip Disease, 94
——— Ulcers of the Tongue, 258
Tuberculosis, 90
Tunbridge Wells Provident Dispensary, 230
Typhoid Fever, 11, 35, 90, 116

Ulster Hospital for Children and Women, 114
Ungrateful Patients, 251
Universities and their Labels, 256
Urinary Tests, 197, 209

Vancouver Hospitals, 89
Venesection in Infantile Disease, 221
Village Drainage, 68
Vivisection, 271
Virchow, American Physicians on, 168

Water, Impure, 90
Waterlow, Sir S. H., 78
Winnepeg General Hospital, 76
Women, The Emancipation of, 110

Year 1891, The, 163

Yellow Fever, 270

### FREE BY POST.

THE COST OF SUBSCRIPTION TO "THE HOSPITAL," post free, is as follows: Per week, 2½d.; per quarter, 2s. 6d.; per half-year, 5s.; per annum, 8s. 6d. Foreign—Per annum, 10s. 8d. Payable, in all cases, in advance.

MONTHLY PARTS, 6d.; post free, 8d. Ditto, per annum, 8s., post free.

CASES FOR BINDING EACH VOLUME may be had of the Publisher, at 1s. 6d. each.

### THE SPECIAL NURSING SUPPLEMENT (MIRROR).

Aberdeen Nurses' Home, xliii
Advertisements, Objectionable, x
All Saints' Nurses, xxiii
Amateurs on Nursing, ci
Ambulance Handbook, An, cvi
American Notes, xvi, xxxi
——— District Nurses, cxxi
——— National Pension Fund, xxxviii
Anniversaries, xciii
Appointments. vi, xvii, xxxv, xxxix, xlvii, liii, lxvi, lxxi, lxxvii, lxxxiii, lxxxviii, xciii, cii, cvii, cxiii, cxxiii, cxxix, cxxxvii, cxl, cxlix, clv
——— Notice of, xc
Army Nurses, cxxi
Asylums, Nurses in, lix
——— Paying Pupils in, xiii
"At Duty's Call," lxxxiii
Australia, Notes from, iv, xxxix, li, lxxiii, cxviii, cxxxv

"Be not Weary," cxii
Bedford Institute, ciii
Belfast News, xliii
Belvedere, Nurses in, xix
Bermuda, A Nurse in, cxxxix
Blind, New Work for the, ciii
Bolton Infirmary, cxlv
Boots and Shoes, lii
Brighton and Hove District Nurses, c
British Nurses' Association, The, cli, cliv
——— Soiree, xlix
Broadstairs Nursing Home, cxxi
Buchanan Cottage Hospital, cix

Canadian School, A, lxxiii, cvi, cix
Cane Hill Asylum, xlix
Canterbury Institution, cxxi
Case Book, Leaves from a Nurses', lxxxi
Certificates, Six Months', vii, xi, xxiii
Children, The Nursing of, xl, ci, cx, cxxx, cxxxiv, cxl, cxlvi, clii
Christmas, lxxxix, xcii, xciv, c, cvii
——— Competitions, xxxiv, xli, lxxii, lxxv
Clarence, Duke of, The Late, xcvii
Clergy and Nurses, xxxvii
Club, A Nurses' Residential, cxlv
Collie, Dr., xxxi
Colney Hatch Asylum, cliv
Concerts in Hospitals, xcii
Cornwall Trained Nurses' Home, cxi
Courage, lxxiii
Cowes, Nursing at, cxiii
Cyprus Society, The, ii, xv
——— A Pupil from, lxxi

Daily Nurses, xliii
Denmark, Nursing in, l
Derby Nursing Sanitary Association, xci
District Nurses, cxxiv, cxlv, cxlix
——— Nursing Association, The, xcvii
Doctors and Nurses, xlix
Dolls, An Appeal for, xi
Dolls, The Nurse, lv, cxxvii
Dress, Nurses', xliv
"Dr. Sutters," xlviii, lx
Dying, Help for the, xxi

East London Nurses, cxxxviii
Eastern Fever Hospital, xxxi
Examination, lxv
——— Questions, xi, xxxiii, lxxxiv, cxix

Freeman, Miss, Memorial to, xli
"Fresh Fields," cliii
Frome Nurses' Home, xcvii
Funerals, cxxiv

Glasgow, A New Home at, lxvii
Glasgow, Grumblers, xxii
——— Nurses, xxxvii, cix, cxv
——— Royal Infirmary, i, xiii, xxxi, lxxvii
——— Sick Poor Nursing Association, lxvii
Guilds, Nursing, cxlv
Gynæcological Nursing, cvi

Halifax Union, i
Hamilton Association, The, vii
Hampshire Institute, The, cxxvii
Hands, Clean, cxxxiii
Her Majesty's Nursing Sisters, lix
Homes for Nurses, cxv
Hospital Scandals, cvi
Hospitals in Towns, lxv
Hysteria, lxvii

"Inasmuch," cxvii
India, Nursing in, lv
Indian Letters, iii, xlvi, lxx, cxii
Indictment, Our, lxxxviii
Infirmary Nursing, xci
Influenza, cxxxix
Insane, Nurses for the, cxlviii
Instruments, Nurses', xix, cxlv
Ipswich Nurses' Home, xxxvii
Irish Lunatics, xxxvii
Irritability, cxi

Ladies, Work for, cli
Lepers, ciii, cxxxiii
Lincoln Hospital, cv
——— Nurses, vii
London Hospital, The, v, lxxvii, clv
——— Hospitals League, The, cli
"Love Divine," ix
Lowestoft Nurses' Association, i

Male Nurses, i, xvii, xxxv
Manchester Nurses, cxxvii
Mashonaland, Nursing in, lxxix
Massage, x
Matrons, Memoranda for, cxxv
Medals and Certificates, l
Medical Women, cxix
Metropolitan Hospital, xli
Monthly Nurses, xi, xxxv
Moral Bacilli, lxxi
Morison Prizes, The, cxviii, cxxxi
Mortality of German Catholic Nurses, The, cxxv
Music as a Soporific, xliii
Musical Boxes in Hospitals, lxv

Natal, A Nurse in, xxii, xxxiv, xliv, lii, lviii, lxxvi, lxxx
Neurasthenic Home, A, lxv
Nightingale Notes, cxv
Norfolk and Norwich Hospital, The, xix, xcvii
North London Nursing Association, cxxiv
Northampton Institution, xliii
Notes and Queries, v, xi, xvii, xxiv, xxxii, xli, xlvii, liii, xlix, lxv, lxxii, lxxvii, xcv, cii, cviii, cxiii, cix, cxxvi, cxxxi, cxxxiv, cxliv, cxlvii, clv
Nurse and Master, cxxvii
——— A Bed for a Sick, viii, xv, xxi, xxxvi, xlii, xlv, liii, lvii, lxvi
——— Training Schools, The, lxxxv
Nurses' Dictionary, The, lxv
——— Co-operation, The, xcvii, cxxxi
——— Cross, The, cvii
——— and Students, v
——— as Patients, x, xxiii
——— Food, v, xvii
——— for the Middle Classes, i, x, xiii, xix
——— Hints to, ii, ci
——— Holidays, lxxix
——— Union, The, ciii
——— Mr. James Payn on, vii, xxiii
Nursing, Amateurs on, ci
Nursing, Notes, lxxv
——— Profession, The, cix

Off Duty, vi, xii, xxiv, xxxvi, lxxii, lxxviii, lxxxiv. xcvi, cii, cviii, cxiv, cxx, cxxvi, cxxxii, cxxxviii, cxliv, cl, clvi

"Pall Mall Gazette," The, and the Hospitals, lxxxviii
Pem Brom Hospital, The, lvi
Perth Nursing Society, cix
Persecuted Hospital, A, clv
Pharmacy to Farming, From, xviii
Poor Law, District Nurses under the, cxxiv
——— Nurses, cxxxiii, cxxxix
Porters, Hospital, cxxxiii
Prescriptions by Correspondence, ciii
Presentations, iv, xvii, xxiii, xxxv, xlii, xlvii, lviii, lxxii, lxxvii, lxxxiv, xc, xcv, cii, cv cvii, cxiii, cxvii, cxxiii, cxxx, cxl, clv
Princess of Wales, The, and the Nurses, ix, l, lvii
Private Nursing, viii
Public, A Lesson for the, cix.

Queen's Nurses, lxxiii, xcii, cxxxvi

Rate-Supported Hospitals, cxv

Reading to the Sick, For, iii, ix, xv, xxi, xxxiii, xxxix, xlv, li, lvii, lxix, lxxv, lxxxii, lxxxvii, xciii, xcix, cv, cxi, cxvii, cxxiii, cxxix, cxxxv, cxlvii, cliii

Red Cross Nurses, xci

Registration, Nurse, cxlvi

Religion and Disease, v

Religious Question, The, xcvii

Reserve, A Nursing, cxxiv, cxxxvii

Rotunda Hospital, Dublin, The, lxxi

Royal National Pension Fund for Nurses, The, lxxxv, cxli

Russian Famine, The, cxv

St. Andrew's Ambulance Association, lxxxv
St. John's Home, lv
St. Mary's Cottage Hospital, lxxi
——— Home, lv, cxxxix
St. Patrick's Home, cxlv
St. Veronica, Guild of, lxvii
Scandals, Real, xciii
Sheffield Workhouse Hospital, cix
Sickness, The Blessings of, li
"Sister is Wanted," cxiii
South Africa, liii
Southend Victoria Hospital, cxxvii
Stockport, Nurses for, xiii
Surgical Ward Work and Nursing, Lectures on, xiv, xx, xxxii, lxviii, lxxiv, lxxxvi, xcviii, civ, cxvi, cxxii, cxxviii.
Survival of the Fittest, The, lvii

Taunton Jubilee Institute, xxxi
Temperature, Local High, xli, lxxvii
Tenby Tittle Tattle, lxvii
Thermometers, xliii.
"To a Nurse," cxlix
Tombstones, xxxi
Training for Nurses, lxxxiii
Trifles, xxi

Ugliness versus Beauty, cl
Uniforms, xiii, cxxxvii, cxli, cxlvii

Vegetarian Dinners, ix, xciv, cvii
Victorian Exhibition, lvii
Village Nursing, xci

Wolverhampton Eye Infirmary, xlix
Worcester Institution, The, cxxxix
Workhouse Nursing, xxxvii, cxxi

York Home for Nurses, cxxvii

Printed by W. H. and L. Collingridge, "City Press," 148 and 149, Aldersgate Street, London, E.C.

### NOTICE TO CORRESPONDENTS.

All MS., Letters, Books for Review, and other matters intended for the Editor should be addressed The Editor, The Lodge, Porchester Square, London, W.

The Editor cannot undertake to return rejected M.S., even when accompanied by stamped directed envelope.

**Notice to Advertisers and Subscribers.**

All Advertisements, Orders for Copies of The Hospital, and Business Communications should be addressed to The Publisher, *not to the Editor*, The Hospital, 140, Strand, W.C.

The Cost of Subscription, post free, is as follows:—Per week, 1½d.; per quarter, 1s. 8d.; per half-year, 3s. 3d.; per annum, 6s. 6d. Foreign:—Per annum, 8s. 8d.; payable, in all cases, in advance.

**NOTICE.—The Index for Vol. IX.. (ending March, 1891) is on sale at the Office of this Journal, price 2½d., post free.**

### General Charities.

[This Column is devoted to an account of work of charitable institutions other than Medical Charities. The Editor will be glad to receive reports or news of work at 140, Strand. *Philanthropy* should be plainly written on the envelope.]

A deficit of two hundred pounds is a serious matter to an institution, the annual income of which barely exceeds that sum. But the usefulness of the **Popular Musical Union** must of necessity be largely curtailed if the close of the present financial year is to be a repetition of the last. The office expenditure of the society is very moderate, and could scarcely be less, and at the same time be productive of efficiency. Notwithstanding financial difficulties, steady progress has been made with the Whitechapel orchestral classes, and it is sincerely to be hoped that a society, which carries on a work which both in its initiation and realisation has had a beneficial influence on the recreations of the industrial classes in London, will not be allowed to languish from want of support.

The cost of the removal of **The School of Discipline** from Chelsea to the new home at Elm House, Parson's Green, owing to the expiry of the lease of the old house, has been more productive of advantages to the children than to the finances of the School, which is still burdened with a small debt that the Committee are anxious to clear. The object of the School, which is the oldest of the kind founded by Mrs. Fry, is the reformation of dishonest, neglected, or destitute female children. Cases for which Female Penitentiaries are provided are inadmissible. Girls, who are received from all parts of England, are admitted between the ages of seven and thirteen years, for a term of not less than two years, but cases sent by a magistrate's order, under the Industrial Schools Act, may be admitted if under the age of fourteen years. The lowest terms for admission are five shillings per week for children whose parents live in London or within eight miles, and six shillings for girls received from places beyond that limit. One of the rules of the School is that there shall be ten different industrial occupations in which all the girls shall be engaged, viz.: Parlour-maids, kitchen-maids, laundry-maids, housemaids, dormitory-maids, schoolroom-maids, clothes girls, orderly monitors, and boot and knife and spoon cleaners. During the past year 47 girls were on the books, 14 of whom were private cases. As each child's maintenance, &c., costs eight shillings, the Committee appeal for continued support from the public.

Dr. Barnardo announces that the "silver wedding fund," the amount of which is to be applied to the reduction of the mortgages upon the buildings connected with his scheme, will not be closed till December 31st. At present nearly £10,000 have been subscribed, and though this sum may be largely augmented during the next three months, it is hardly probable that the sum of £50,000, which is necessary for the extinction of the mortgages will be reached. During the past emigration season, which closed in August, 373 boys were sent out to Canada and the Colonies, 106 having left on August 19th last. No fewer than 63 of this latter party had, it is stated, at one time or another been actually homeless on the streets, and nearly half were entirely orphaned. The whole of the cost of this party was defrayed by the generosity of a single donor, but the expenses of some forty boys who sailed in June last have not yet been defrayed. In order to remove this burden the sum of £400 is required, as the average cost of sending a boy to Canada is £10. The indefatigable Director of the Homes has resolved to extend his work in a new direction. It appears that for a long time he has continued to receive numerous applications for advice and assistance from the parents of boys who are not destitute but who are described under the somewhat wide term of "troublesome." We can hardly imagine the existence of such a prodigy as the boy who is never troublesome. But it would seem that the word is used in its worst sense, as applying to boys whose misconduct is such as to cause grave fear for their future should they continue in their usual surroundings. As no comprehensive scheme now exists for the benefit of boys of this class, Dr. Barnardo has resolved to open a special branch, which will deal with boys of a superior class to that which furnishes most of the candidates for admission to the Homes. The limit of age will be from twelve to fifteen years, and every lad will be bound to the Institution for three years' training. No boys will be taken who are not in good physical health and of at least average intelligence. "The principle to be adopted," to quote Dr. Barnardo, "will be that of constant and interesting occupation for every wakeful hour, such occupation to be in some manual employment, in educational pursuits, or in sports, so as to leave no leisure for loafing or mischief." By this means, and by inculcating discipline similar to that adopted in the Royal Navy, Dr. Barnardo hopes to build up gradually a thoroughly healthy and honourable tone and character among the boys. Payment will be required in every case, the scale to be determined by circumstances; but it will not be less than £25 or more than £50, and the training received by the lads will be for a seafaring and colonial life. Dr. Barnardo invites communications from parents and guardians of "troublesome" boys, which should be addressed to Mr. John Odling, 18 to 26, Stepney Causeway, London, E.

### Scraps and Gleanings.

Hospital Saturday at Watford produced £155.

The annual soiree at St. Thomas's was held on Wednesday.

The Mayor of Cork will inaugurate the first Hospital Saturday in that town.

Another of the lady medical students at the Royal Free is engaged to a doctor.

A small rustic lately described a skeleton as "a man with the meat off him."

Dr. William Henry Viner committed suicide at South Bermondsey last week.

The new operation theatre at the London was christened on Friday by Mr. Treves.

A dealer in artificial limbs estimates that 30,000 Britons have lost one or both legs.

Dorset County Hospital will benefit by about £30 from Weymouth Hospital Sunday.

Ramsgate and St. Lawrence Royal Dispensary treated 2,166 patients last year; funds are satisfactory.

Mrs. Leith Adams, the novelist, held a flower stall at Stratford-on-Avon on Hospital Saturday.

In *Good Words* for October are some verses called "A House Surgeon's Story," which are well worth reading.

Mansfield and Mansfield Woodhouse Hospitals are in future to be under separate Matrons. Miss Pell Smith has resigned.

The ladies of Bury are to be congratulated on having collected £81 for the Suffolk General Hospital, being £11 more than last year.

A competitive fasting performance to last sixty days is being arranged at New York. Five competitors in starvation will begin on October 5th.

The Chief of Gondal, India, who studied medicine in Edinburgh some years ago, maintains six hospitals in his district which last year treated 50,000 patients.

The Bolton Rural Sanitary Board paid a visit to their infectious hospital lately, and found all in excellent order and the wards looking bright and cheerful.

The death is announced of Elizabeth Denkin, at Penrith, at the age of 102. She was blind and deaf for several years. Her surviving daughter is over 70 years of age.

The Secretary for War has approved of Surgeon-Captain H. R. Whitehead, Medical Staff, being appointed Assistant Professor of Surgery at the Army Medical School, Netley.

Mrs. Ashton Warner is leaving the East-end Mothers' Home, which she helped to found, and means to emigrate to America with her children. The good wishes of many go with her.

The inspector reports that the inmates of the Peterboro' Infirmary are not unkindly treated, and that the reports lately circulated by Mr. Deane are sensational and exaggerated.

The Committee of Bristol General Hospital at their half-yearly meeting bewailed the lack of funds wherewith to go on with their building, though the hospital was crammed full.

The Governors of the Bradford Infirmary have unanimously confirmed the action of the committee in arranging to take over the management of the Woodlands Convalescent Home at Rawdon.

The rules of the Inverness Infirmary are being materially altered. In future preference will be given to a Medical Officer who has had previous experience, and much of the clerical work which fell on the late Matron will be handed over to a clerk.

The compulsory application of the Notification of Infectious Diseases Act with its corollary of an infectious hospital is, as Mr. Ritchie explained at the luncheon held in honour of the inauguration of the Mortlake Sewage Works, to be carried into law as speedily as may be.

The Lanark Hospital reports a deficit of £99, and appeals for support. It is a pretty little hospital, nursed by the Sisters of St. Vincent de Paul, whose management is most economical. Being near such a rich town as Glasgow it ought to find friends rich enough to come to its aid.

Sunderland Infirmary report is just out. It states that during the year 2,159 in-patients were treated and 2,701 out-patients. The expenditure was £7,861; the workmen contributed over £4,000 to the income of the hospital. This speaks well for the popularity of the institution.

The small-pox epidemic in Leeds shows no signs of decreasing; in fact, appearances point in the opposite direction. There have been many new cases lately. One of these came from Headingley, a fashionable suburb which has hitherto escaped the infection. The new Sanatorium in York Road is progressing towards completion.

Two more anti-vaccinationists have been fined at Bishops Stortford, and at Chelmsford the anti-vaccinationists are paying dearly for their principles. A few days ago the goods of the Rev. J. M. Whiteman and two others were distrained upon, but the articles did not realise the full amount of the fines and costs, and it seems that another distraint will be levied.

Under the auspices of the Local Friendly, Trade, and Temperance Societies' Committee, a very successful Chinese lantern parade was held in Stratford on Saturday in aid of West Ham Hospital, the original suggestion for the formation of which sprang from the committee in question. The procession was a picturesque one, and attracted great attention.

### THE STUDENT OF MEDICINE.

[All communications for this Supplement should be addressed to The Editor of The Hospital, at 140, Strand, with the words, "Students' Column," written in the left hand top corner of the envelope.]

### APPOINTMENTS.

At the Cheshire County Asylum, Macclesfield, J. A. Cooke, M.R.C.S., L.R.C.P., has been appointed junior assistant-medical officer. At the Coventry and Warwick Hospital, C. Frier, of Cupar, N.B., has been appointed assistant house surgeon. At the Stourbridge Dispensary, J. S. Maynard, M.B., C.B.Edin., has been appointed house surgeon and secretary. At the Rotherham Hospital, L. J. Weatherbe, M.B., C.M.Edin., has been appointed house surgeon. At the Salford Royal Hospital, R. E. Lord, M.B., B.Sc. (London), M.B., C.M. (Victoria), M.R.C.S., who has occupied the position of junior house surgeon, has been elected senior house surgeon; and Charles Christopher Heywood, M.A., M.B., B.S. (Cantab), M.R.C.S., L.R.C.P., L.S.A., has been elected to fill the office of junior house surgeon.

### VACANCIES.

The following vacancies are announced this week, the amount of salary in each case being given after the name of the institution:

**Resident Medical Officers.**

Bethlem Hospital, S.E., Two Resident Clinical Assistants.
Cheshire County Asylum, Upton, near Chester, Junior Assistant Medical Officer—Salary £120.
South Devon and East Cornwall Hospital, Plymouth House, Surgeon—Salary £100.
Walsall Cottage Hospital, Resident House Surgeon—Salary £100.

**Non-Resident Medical Officers.**

Dental Hospital of London, Leicester Square, Anæsthetist and Assistant Anæsthetist.
Middlesex Hospital, Pathologist and Curator of the Museum
University of Aberdeen, Six Examiners in Medicine—Grant of £30.
West Kent General Hospital, Maidstone, Honorary Dental Surgeon.

### PASS LISTS.

**Royal Colleges of Physicians and Surgeons.**

The following gentlemen passed the first examination of the Board in materia medica and pharmacy at the quarterly meeting of the examiners:—Messrs. E. W. Adams, private study; P. E. Adams, private study; H. G. S. Anderson, students of Owens College, Manchester; A. Armer, private study; W. C. G. Ashdowne, St. Mary's Hospital; W. S. Aslett, private study; G. E. Atkins. private study; E. D. Aubin, Middlesex Hospital; T. W. N. Barlow, University College. Liverpool; W. C. Beddard, St. Thomas's Hospital; H. T. S. Bell, Guy's Hospital; A. G. Bennett, St. Mary's Hospital; D. R. N. Bernhardt, St. George's Hospital; J. W. Bird, Guy's Hospital; J. R. Bishop, Owens College, Manchester; P. L. Blaber, St. Thomas's Hospital; A. F. Blake, London Hospital; T. W. W. Bovey, Bristol Medical School; A. J. H. Boyton, private study; F. J. Brakenbridge, St. Thomas's Hospital; G. W. Brown, St. Thomas's Hospital; H. O. B. Browne-Mason, St. Mary's Hospital; F. M. Burnett, St. Bartholomew's Hospital; P. D'E. Burrell, St. George's Hospital; H. A. Burridge, private study; W. J. Burroughs, Guy's Hospital; L. W. Burrow, St. Thomas's Hospital; W. Butler, Guy's Hospital; W. F. Byford, Guy's Hospital; J. E. G. Calveley, St. Bartholomew's Hospital; A. Cant, Queen's College, Birmingham; H. Martin Case, Middlesex Hospital; J. S. Chater, private study; A. Hopkins Clarke, St. Bartholomew's Hospital; G. B. Clarke, Charing Cross Hospital; C. C. Clarkson, University College; E. T. Coady, private study; A. W. R. Cochrane, private study; C. J. E. Cock, Charing Cross Hospital; W. R. Coldicott, Charing Cross Hospital; E. Coleman, Guy's Hospital; J. Jordan Coleman, Guy's Hospital; F. S. Collard, St. George's Hospital; C. R. Colley, Guy's Hospital; G. W. Connor, Middlesex Hospital; A. H. Copeman, St. Thomas's Hospital; S. Copley, Guy's Hospital; C. George Cory, St. Bartholomew's Hospital; G. A. Crace-Calvert, St. Bartholomew's Hospital; R. F. W. Crawford, St. George's Hospital; F. G. Crookshank, University College; H. C. Crouch, St. Thomas's Hospital; J. A. Crump, private study; A. P. Cummings, Yorkshire College, Leeds; J. Currie, St. Bartholomew's Hospital; J. E. P. Davies, St. Mary's Hospital; J. A. Dawes, Owens College, Manchester; R. L. Dickinson, London Hospital; A. Dimsey, University College; C. H. Dissent, University College; W. E. Dickson, St. Thomas's Hospital; E. J. Dobbin, Middlesex Hospital; E. A. Dorrell, Bristol Medical School; E. Down, private study; E. G. D. Drury, St. Bartholomew's Hospital; R. Everett Duke, University College; C. R. Dykes, private study; H. F. Ealand, St. Mary's Hospital; P. J. Edmunds, University College; J. A. Edsell, St. Bartholomew's Hospital; R. M. Ellis, St. George's Hospital; H. B. Emerson, Yorkshire College, Leeds; C. A. Ensor, Guy's Hospital; C. C. J. Erhardt, King's College; A. G. Ewbank, St. Bartholomew's

### THE STUDENT OF MEDICINE—(*continued*).

Hospital; C. H. Fagge, Guy's Hospital; W. E. Fairweather, Owens College, Manchester; R. W. Fisher, St. Bartholomew's Hospital; E. Folliott, St. Bartholomew's Hospital; W. D. Frazer, St. Thomas's Hospital; A. C. Friend, St. Thomas's Hospital; W. Gabe, Middlesex Hospital; G. F. S. Genge, Westminster Hospital; G. G. Geng[illegible], St. Thomas's Hospital; A. P. Gibbons, London Hospital; A. H. Godwin, University College, Liverpool; E. G. L. Goffe, University College; F. Golding-Bird, Guy's, Hospital; A. F. Goldsmith, St. George's Hospital; J. E. Gordon, Glasgow University; E. H. G. Grabham, St. Thomas's Hospital; W. H. Gray, University College; T. H. Green, Guy's Hospital; G. F. Gregory, Bristol Medical School; J. M. K. Grover, Owens College, Manchester; E. Haines, St. Thomas's Hospital; E. S. Hall, Guy's Hospital; R. J. E. Hanson, St. Mary's Hospital; W. Hardcastle, Charing Cross Hospital; F. H. Hardy, Guy's Hospital; T. Herbert, Middlesex Hospital; W. Herbert, St. Thomas's Hospital; C. M. Hewer, St. Bartholomew's Hospital; J. C. Hibbert, University College; W. C. D. Hills, Charing Cross Hospital; W. W. Hoare, Edinburgh University; A. L. Home, St. Thomas's Hospital; W. E. L. Horner, University College; A. C. Hovenden, Guy's Hospital; G. S. Hovenden, Guy's Hospital; A. O. Howse, London Hospital; W. E. Huddleston Middlesex Hospital; B. M. Hughes, St. Bartholomew's Hospital; R. Hughes, St. Thomas's Hospital; J. H. Hugo, private study; A. Hunnard, University College; G. B. Hunt, University College; H. Jackson, Yorkshire College, Leeds; F. H. Jacob, King's College; S. P. James, St. Mary's Hospital; A. W. Jenkins, University College; R, M. Johnston, Univer-College; G. B. Kaufmann, London Hospital; R. Kay, Guy's Hospital; C. J. Kearney, Guy's Hospital; P. W. Kent, St. Thomas's Hospital; D. B. Keown, St. Bartholomew's Hospital; A. F. W. King, St. Thomas's Hospital; P. Kitchin, Yorkshire College, Leeds; J. P. Kitson, University College; F. C. Langford, private study; H. G. Lawrence, St. Mary's Hospital; F. G. Layton, St. Thomas's Hospital; T. P. Legg, St. Bartholomew's Hospital; T. R. Llewellyn, University College; H. W. Lloyd, University College, Liverpool; M. W. Loy, London Hospital; E. J. Lumb, Yorkshire College, Leeds; A. A. Macfarlane, St. Bartholomew's Hospital; F. B. Madden, St. Bartholomew's Hospital; F. R. Mann, Middlesex Hospital; M. Marriott, Sheffield Medical School; G. S. S. Marshall, Middlesex Hospital; F. H. L. Marson, Middlesex Hospital; A. K. Matthews, Guy's Hospital; W. S. Mayne, University College; J. H. Meacher, St. Bartholomew's Hospital; Q. H. Miller, Charing Cross Hospital; H. W. Mills, St. Thomas's Hospital; E. Morris, private study; J. Moses, London Hospital; C. S. Murray, St. Mary's Hospital; R Newnham-Davis, St. Mary's Hospital; W. P. Nichol, Queen's College, Birmingham; H. W. Nott, University College, Liverpool, J. H. F. Nunn, private study; A. L'E. Orme, Dublin; K. J. P. Orton, St. Thomas's Hospital; W. J. Owen, University College, Liverpool; H. D. Packer, Guy's Hospital; W. C. Pakes, private study; M. H. C. Palmer, private study; C. C. Parsons, St. Mary's Hospital; M. S. Paterson, St. Mary's Hospital; W. H. J. Paterson, St. Thomas's Hospital; F. J. Pearson, private study; M. Grey Pearson, St. Bartholomew's Hospital; B. F. Pendred, Guy's Hospital; L. N. Pentreath, St. Thomas's Hospital; A. E. Phillipps, Guy's Hospital; G. Phillips, University College; A. A. Price Guy's Hospital; H. J. Price, University College; P. J. Probyn, Charing Cross Hospital; C. S. Read, University College; R. B. Rees, Middlesex Hospital; G. F. Reynalds, St. Barthomew's Hospital; B. G. Reynolds, Guy's Hospital; J. Richards, University College; R. L. Roberts Guy's Hospital; W. Roberts, University College; J. Robertson, Guy's Hospital; C. H. J. Robinson, Middlesex Hospital; J. C. R. Robinson, St. Thomas's Hospital; A. A. Rogers, private study; W. J. Rowland, Guy's Hospital; W. H. Rowthorn, Sheffield Medical School; F. J. Sadler, Oxford University; L. J. Salter, private study; O. Sapara, private study; A. L. Saunders, St. Bartholomew's Hospital; H. S. Schultess-Young, Westminster Hospital; A. E. Sears, London Hospital; W. A. Sharpin, St. George's Hospital; S. E. Shoppee, University College; J. C. Smellie, St. Mary's Hospital; C. W. Smith, University College; G. U. Smith, King's College; G. H. Sowry, St. Bartholomew's Hospital; T. A. Starkey, University College; J. R. Steinhaeuser, Guy's Hospital; H. J. Stevens, Charing Cross Hospital; H. B. Stoner, Guy's Hospital; A. C. Strand, Middlesex Hospital; R. G. Strange, St. Thomas's Hospital; F. C. Sutherland, St. Bartholomew's Hospital; S. E. Tench, Middlesex Hospital; D. L. Thomas private study; E. R. Thomas, Guy's Hospital; R. T. Thomas, University College; W. G. Thomas, Queen's College, Birmingham; W. J. Thomas, Middlesex Hospital; W. P. Thomas, London Hospital; H. E. Thompson, private study; H. C. Thornton, St. Thomas's Hospital; E. O. Thurston, St. Thomas's Hospital; F. L. Titley, Bristol Medical School; H. G. Toombs, St. Thomas's Hospital; P. E. Tresidder, Guy's Hospital; A. C. Turner, Middlesex Hospital; H. F. Turner, Guy's Hospital; T. W. Turner, London Hospital; P. Twomey, Owen's College, Manchester; E. H. Van Someren, Guy's Hospital; J. N. B. Vise, Westminster Hospital; J. Wallwork, Owens College, Manchester; P. L. Watkin-Williams, Middlesex Hospital; A. L. A. Webb, University College; C. C. Weeks, University College; T. H. Wells, Middlesex Hospital; R. Wheatley, Middlesex Hospital; M. Wheeler, St. Thomas's Hospital; A. B. Whishaw, St. Thomas's Hospital; F. J. Whitehead, Queen's College, Birmingham; F. Whitelaw, St Mary's Hospital; J. H. Wigham, Yorkshire College, Leeds; T. H. Wilkins, Queen's College, Birmingham; T. H. Wilkins, Charing Cross Hospital; M. Wilks, University College, F. J. I. Whilley, Melbourne University; J. D. E. Williams, St. Thomas's Hospital; W. M. Willis, Bristol Medical School; F. W. Willway, Bristol Medical School; H. M. Wise, Guy's Hospital; H. Witham, Westminster Hospital; W. B. Woodhouse, Middlesex Hospital; A. Woodward, St. Bartholomew's Hospital; L. Worts, Guy's Hospital; and W. E. Wyborn, Charing Cross Hospital.

